# Rich Get Richer: Extraversion Statistically Predicts Reduced Internet Addiction through Less Online Anonymity Preference and Extraversion Compensation

**DOI:** 10.3390/bs12060193

**Published:** 2022-06-16

**Authors:** Shaozhen Zhang, Wenliang Su, Xiaoli Han, Marc N. Potenza

**Affiliations:** 1 Department of Applied Psychology, School of Humanities and Social Sciences, Fuzhou University, Fuzhou 350108, China; 202920017@fzu.edu.cn (S.Z.); n192920010@fzu.edu.cn (X.H.); 2 Department of Psychiatry, Child Study Center, Department of Neuroscience, Yale University School of Medicine, New Haven, CT 06510, USA; marc.potenza@yale.edu; 3 Connecticut Mental Health Center, New Haven, CT 06519, USA; 4 Connecticut Council on Problem Gambling, Wethersfield, CT 06109, USA; 5 Wu Tsai Institute, Yale University, New Haven, CT 06520, USA

**Keywords:** extraversion, online and offline integration, anonymity, extraversion compensation, internet addiction, rich get richer

## Abstract

Internet addiction may arise from multiple factors and personality tendencies have been previously implicated. Prior studies have found that extraversion may be a protective factor mitigating against internet addiction, yielding a “rich-get-richer” effect. However, few studies have explored how extraversion may influence internet addiction from the perspective of online-offline integration. Drawing on a sample of 428 college students, the current study examined a serial mediation model exploring the underlying mechanisms of how extraversion may statistically predict internet addiction through online-offline integration and antecedent factors. The serial mediation model analyses indicated that extraverted internet users exhibited a weaker preference for online anonymity and less online extraversion compensation, thus formulating a higher level of online-offline integration than introverted individuals, which, in turn, appeared to reduce the risk of internet addiction. In contrast, with regard to specific components of online-offline integration, introverted internet users preferred online anonymity, which reduced their relationship integration and increased their likelihood of internet addiction; similarly, the introverted individuals were also more likely to exhibit an extraversion compensation effect. That is, they were more extraverted on the internet than in general; hence, they had a lower level of self-identity integration, resulting in a greater likelihood of experiencing internet addiction. These results highlight the importance of online-offline integration that may account for personality variations in social and psychological outcomes related to internet use, and suggest a role for online anonymity preference and extraversion compensation in influencing specific components of integration.

## 1. Introduction

While the evolving internet brings convenience, it also brings a series of concerns, including internet addiction. Internet addiction has been defined as “use of the internet that creates psychological, social, school, and/or work difficulties in a person’s life” [1]. In addition to generalized internet addiction, researchers have also described specific types of internet addiction, such as social media addiction, internet gaming disorder, and pornography addiction [2,3,4]. Internet addiction may negatively impact people in different ways. In the short term, internet addiction may lead to poor sleep and lower academic performance [5,6]. In the long run, people with internet addiction appear to be at an increased likelihood of experiencing depression and other psychological problems [7]. Considering the prevalence of internet use, many researchers have been studying internet addiction and its associated factors.

Like other addictions, internet addiction has a complex etiology with multiple contributing factors implicated. According to a biopsychosocial model of addiction [8], the development of internet addiction may involve contributions from neurobiological (e.g., genetics and neurotransmitters) [9,10], more stable psychological (e.g., personality features, impulsivity, inhibitory-control tendencies) [11,12,13], or more state-related psychological (e.g., stress, loneliness) factors [14,15], as well as factors related to social environment (e.g., social norms, family, school) [16,17]. Of these, personality tendencies have received considerable attention, in that they may influence risk for internet addiction [18,19]. Among the personality factors, extraversion and its opposite, introversion, are considered important [20]. Several meta-analyses have found that extraversion was negatively related to internet addiction [12,21]; however, a weak positive association between extraversion and smartphone addiction was identified in another meta-analysis [22]. Available results on associations between extraversion and internet addiction are seemingly inconsistent, resonating with two rival theoretical hypotheses that aim to explain who may benefit from internet use: the rich-get-richer or social enhancement hypothesis and the poor-get-richer or social compensation hypothesis.

The “rich-get-richer” hypothesis proposes that people with higher extraversion or lower social anxiety are better at using the internet as another mechanism to build their social circles, so extraverted individuals can make more friends online than introverted ones, leading to better mental health outcomes [23,24]. On the other hand, the social compensation hypothesis proposes that introverted individuals or those with less self-esteem may gain more from bridging social capital when using the internet [25]. Introverted individuals may feel more comfortable interacting with others online as they can carefully consider how to respond to others instead of feeling nervous and distressed in offline social interactions. In other words, the lack of visual cues and the perceived anonymity of the internet environment may help introverted or socially anxious individuals to overcome their inhibitions and to get into contact with others [26,27,28]. As such, rich-get-richer and social compensation are two opposing hypotheses that may support different predictions of how extraversion may relate to internet addiction. Given that both hypotheses have gained empirical evidence, it is possible that they are both valid [29,30]. Some researchers have suggested that personality features do not directly influence the outcomes people receive from utilizing online services, but they may increase the likelihood of specific behaviors that may increase the likelihood of certain outcomes, which is a phenomenon described as a modified rich-get-richer effect [28,31]. However, few studies have systematically investigated kinds of internet behaviors and motivations with respect to social and psychological outcomes.

To consider how to make the best use of the internet and address the conflict between “rich-get-richer” and social compensation hypotheses, Lin, Su, and Potenza [32] proposed the online and offline integration hypothesis (termed the “integrated-get-richer” hypothesis in the current study), suggesting an increase in online-offline integration may promote balance and coordination between the internet space and real life, which may in turn promote rational use of the internet and prevent problematic use. Suler also noted that merging online and offline lives could contribute to enhancing individuals’ development and success [33]. The “integrated-get-richer” hypothesis may help explain findings differentially supporting the “rich-get-richer” and social compensation hypotheses, and it may provide direction on questions that the modified “rich-get-richer” hypothesis has not fully addressed [31]. It is possible that extraverted individuals are generally more likely to have a higher integration level of internet use and thus benefit more from using the internet. Considering that there are more studies on personality and generalized internet addiction and that internet addiction may be considered to be an “umbrella” term encompassing multiple specific internet addictions [34], we chose to first study the relationships between extraversion and internet addiction. In line with the previous study [32], we put forward the hypothesis that:

**Hypothesis** **1** **(H1).** *Online-offline integration will mediate the negative association between extraversion and internet addiction*.

Whereas online-offline integration may influence associations between extraversion and internet addiction, the question of how extraversion influences an individual’s integration tendency remains unanswered. To illuminate, antecedent factors linked to integration need to be addressed. One possible factor may be a preference for online anonymity. Online communications perceived as anonymous in virtual contexts may allow introverted individuals to feel more confident when interacting with friends [35]. Online anonymity offers the possibility of identity construction from which people can explore different aspects of their identities [36]. Introverted individuals may have a stronger preference for online anonymity to improve their self-presentation than extraverted ones. Thus, anonymity may hinder individuals’ online-offline integration, especially in relationships. Furthermore, anonymity is one etiologic factor contributing to internet addiction according to the ACE model [35], and the appeal of an anonymous environment may result in an over-reliance on online interpersonal relationships and lead to internet addiction [37]. Taken together, we put forward the hypotheses that:

**Hypothesis** **2** **(H2).** *Preference for online anonymity will mediate the association between extraversion and online-offline integration*.

**Hypothesis** **3** **(H3).** *Preference for online anonymity will positively predict internet addiction*.

Surveys suggest that people may express different personalities online and offline, including with respect to extraversion [38]. Introverted individuals may be empowered by the internet, allowing them to experience less inhibition and be more outgoing, social, and involved than in face-to-face situations [39,40]. Thus, the introverts may exhibit greater extraversion online than offline, which can be termed an extraversion compensation effect. With such effects, personality differences may be differentially expressed between online and offline environments, thus decreasing the level of self-identity integration proposed by the “integrated-get-richer” hypothesis [32]. It was also found that participants with internet addiction reported higher scores in extraversion online than in general, namely showing the extraversion compensation effect [41]. Based on these possibilities, we proposed that:

**Hypothesis** **4** **(H4).** *Extraversion compensation may play a mediating role between extraversion and online-offline integration*.

**Hypothesis** **5** **(H5).** *Extraversion compensation will be negatively related to internet addiction*.

**Figure 1:**
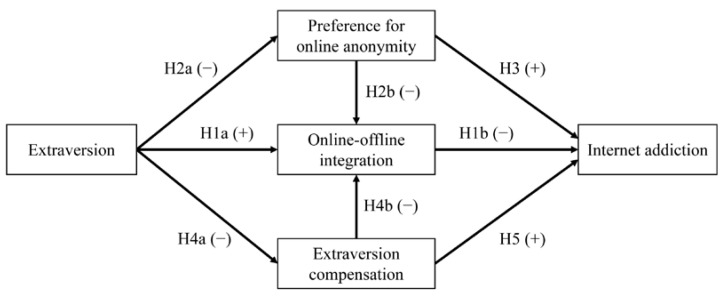
Hypothesized model proposed in this study. + hypothesized positive influence; − hypothesized negative influence.

Furthermore, online-offline integration involves three important aspects, including self-identity, relationships, and social function [32]. Therefore, we were interested in exploring whether the preference for online anonymity and extraversion compensation may play different roles in mediating associations between extraversion and internet addiction through different domains of online-offline integration (**Research Question 1**). In sum, the research concept model is illustrated in Figure 1.

## 2. Methods

### 2.1. Sample and Procedure

Five-hundred Chinese college students from Fuzhou University volunteered to be recruited to the study and they anonymously completed questionnaires using paper and pencil offline. However, some participants were excluded (n = 72) as they only completed part of the scales (n = 48) or reported the same values for all items within the questionnaire in multiple scales (n = 24), raising questions regarding their veracity. As a result, the remaining 428 valid responses were used for further analysis. The final sample had a mean age of 20.33 years (SD = 1.77, range 15 to 29) and contained 201 (40.7%) men. The sample size in this study was planned to be larger than the recommended minimum sample size (N = 200) for SEM as a rule of thumb [42], considering the complexity of the model (N = 5−10 per estimated parameter) [43,44]. The valid sample size was similar to those reported in previous studies [28,45].

### 2.2. Measures

#### 2.2.1. Preference for Online Anonymity Questionnaire

We created a 10-item questionnaire to assess participants’ preferences about online anonymity (i.e., ‘‘I prefer to anonymously express different opinions to others on the internet.’’ For more items, see the Appendix A). Participants responded to each item on a 5-point Likert-like scale (1 = strongly agree, 5 = strongly disagree) and a higher score indicated a stronger preference for perceived anonymity on the internet. The scale had sound construct validity (factor loadings in the exploratory factor analysis ranged from 0.43 to 0.78) and acceptable internal consistency (Cronbach’s α = 0.77). 

#### 2.2.2. The Online and Offline Integration Scale (OOIS)

Participants’ levels of online and offline integration were measured using the OOIS questionnaire [32]. The OOIS has 3 subscales, each with 5 items, including self-identity integration (Cronbach α = 0.75, e.g., “My online and offline selves look like two completely different people”, reverse item), relationship integration (Cronbach α = 0.71, e.g., “On the internet, I mostly interact with my offline friends or family members”), and social function integration (Cronbach α = 0.66, e.g., “Most of my online activities serve the needs of my study, job or daily life”). Participants used a four-point Likert scale to answer the items (1 = strongly disagree, 2 = disagree, 3 = agree, and 4 = strongly agree). The overall scale’s reliability coefficient was 0.77. After taking account of the reverse coding, the three subscales’ scores were summarized as total integration scores, with higher scores indicating higher levels of online-offline integration.

#### 2.2.3. Internet Addiction Test (IAT)

The IAT was used to measure participants’ dependence on the internet, which comprised of 20 questions with five-point Likert responses (1 = rarely, 5 = always) [37]. The total score ranged from 20 to 100, with higher scores reflecting more severe levels of internet addiction. Participants who scored 20–49 were considered to have average internet use, 50–79 indicated a moderate level of internet addiction, and scores of 80–100 indicated severe internet addiction. Prior studies have shown the IAT to be a valid and reliable instrument [46,47]. In this study, the Cronbach α was 0.89.

#### 2.2.4. Extraversion and Online Extraversion Compensation

The self-report questionnaire Eysenck Personality Questionnaire-Revised, Short Scale for Chinese (EPQ-RSC), revised by Qian, Wu, Zhu, et al. [48], has been widely utilized in China for clinical and research purposes. The EPQ-RSC includes four subscales related to extraversion, neuroticism, psychoticism, and lying. 

In the current study, extraversion was measured by the 12-item extraversion subscale. Items (e.g., “Are you a talkative person?”) were answered with “yes” or “no” responses depending on the applicability of the statement. Higher scores suggest greater extraversion in general. Cronbach’s α was 0.80 in this study, indicating good internal consistency. 

Referring to the previous practice [38,41], we also measured participants’ online extraversion with the modified 12 items of the extraversion subscale of the EPQ-RSC by adding “on the internet” to each item (e.g., “Are you a talkative person on the Internet?”). Similarly, higher scores reflect greater extraversion on the internet. These items also showed good reliability in the study (Cronbach’s α = 0.79). 

To reflect the level of online extraversion compensation effect, we subtracted the scores of the (general) extraversion from the online extraversion. Therefore, positive scores reflect higher extraversion online than generally.

### 2.3. Statistical Analysis

Missing data for all variables were handled with mean imputation because the proportions were very small (less than 1%) [49]. Pearson correlation analysis was performed, using SPSS 20.0 software, to evaluate relationships between variables. Structural equation modeling (SEM) analyses and bootstrap methods were conducted, using Mplus7.0, to test mediating effects within the model. Additionally, model fit was evaluated using fit indices, including the root mean square error of approximation (RMSEA), the standardized root mean square residual (SRMR), the comparative fit index (CFI), and the Tucker−Lewis fit index (TLI). The model was considered to be an excellent model, with the values of RMSEA and SRMR less than 0.06 and the value of CFI and TLI more than 0.95 [50].

## 3. Results

### 3.1. Preliminary Analyses

According to the results of the IAT scores, 54.2% of participants had average internet use while 44.6% were considered as having moderate internet addiction and 1.2% were considered as having severe internet addiction. In addition, means, standard deviations, and correlation analysis were performed for each variable (Table 1). Extraversion was negatively associated with a preference for online anonymity, extraversion compensation, and internet addiction, and positively related to online-offline integration. In addition, online-offline integration was negatively related to a preference for online anonymity, extraversion compensation, and internet addiction. More results regarding the dimensions of integration are shown in Table 1.

### 3.2. Testing for Mediating Effects

**Figure 2:**
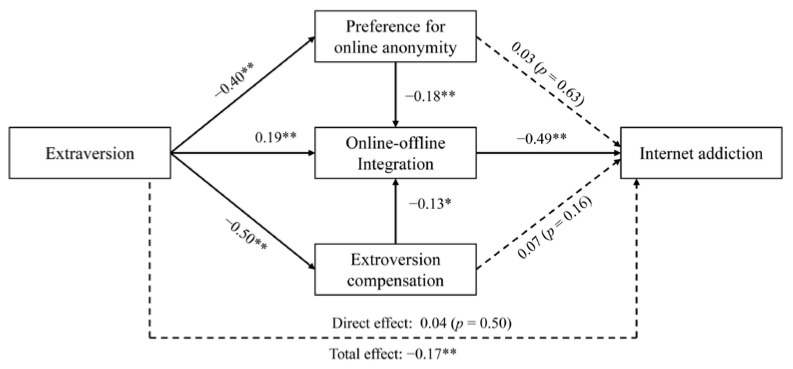
Results of the serial mediation model from extraversion to internet addition. Coefficients are standardized betas, * *p* < 0.05, ** *p* < 0.01.

SEM analyses (5000 bootstrap resamples) were conducted to test the mediating effects in the hypothetical model. As shown in Figure 2, the total effect of extraversion on internet addiction was significant (β = −0.17, *p* < 0.01), but after controlling for a preference for online anonymity, extraversion compensation, and online-offline integration, the direct effect of extraversion on internet addiction was not significant (β = 0.04, *p* = 0.50). Specifically, extraversion positively predicted online-offline integration (β = 0.19, *p* < 0.01), and integration negatively predicted internet addiction (β = −0.49, *p* < 0.01); therefore, H1 was confirmed. Extraversion also negatively predicted a preference for online anonymity (β = −0.40, *p* < 0.01), which, in turn, negatively predicted online-offline integration (β = −0.18, *p* < 0.01); thus, H2 was supported. Whereas H3 predicted that a greater preference for online anonymity would relate significantly to internet addiction, we found this direct effect was not as significant as expected (β = 0.03, *p* = 0.63), but it was indirectly mediated by online-offline integration. In line with H4, extraversion compensation mediated the relationship between extraversion and online-offline integration (β = −0.50, *p* < 0.01; β = −0.13, *p* = 0.01). However, extraversion compensation did not relate directly to internet addiction as predicted in H5; instead, this relationship was mediated by online-offline integration. Taken together, a preference for online anonymity and online-offline integration sequentially mediated the relationship between extraversion and internet addiction; similarly, extraversion compensation and online-offline integration also sequentially mediated the relationship. The bootstrapping estimate effects of both direct and indirect paths are shown in Table 2. The model’s fitting result indicated an excellent model fit, RMESA < 0.001, SRMR = 0.003, CFI = 1.00, TLI = 1.02. 

To further examine Research Question 1, relating to the different roles of preference for online anonymity and extraversion compensation on the three components of online-offline integration, the variable of online-offline integration was replaced with its three dimensions in the new model. Furthermore, the direct paths of “preference for online anonymity → internet addiction” and “extraversion compensation → internet addiction” were trimmed as they were not significant in the prior model. Result shows that the new model has a good fit: RMESA = 0.016; SRMR = 0.014; CFI = 0.999; TLI = 0.996. 

**Figure 3:**
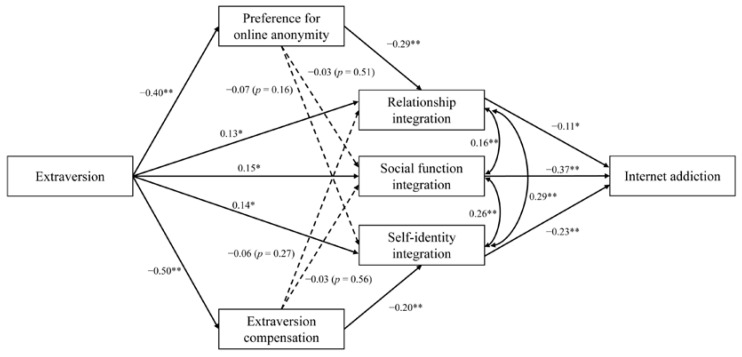
Results of the serial mediation model with specific integration components. Coefficients are standardized betas, * *p* < 0.05, ** *p* < 0.01.

As shown in Figure 3, the results suggest that all three components of online-offline integration could mediate the influence of extraversion on internet addiction. In addition, the results show that a preference for online anonymity mediated the relationship between extraversion and relationship integration; similarly, the influence of extraversion on self-identity integration was mediated by extraversion compensation. Therefore, preference for online anonymity and relationship integration could sequentially mediate the link between extraversion and internet addiction, and so could extraversion compensation and self-identity integration.

## 4. Discussion

Personality is a highly relevant factor in determining behavior on the internet [51]. This study provides new insight into how extraversion may lead to internet addiction from the perspective of the “integrated-get-richer” hypothesis. We found that a preference for online anonymity, extraversion compensation, and online-offline integration (as well as its dimensions) serially mediated the relationship between extraversion and internet addiction. The results highlight the importance of online-offline integration that may account for some variations in social and psychological outcomes related to internet use, and reveal the role of preference for online anonymity and extraversion compensation on specific components of online-offline integration, which may have important theoretical and practical values for understanding and promoting a healthy use of the internet. 

The “rich-get-richer” and the social compensation hypotheses are valuable to gain a better understanding of the types of people (e.g., extraverted or introverted) who benefit from internet use [24]. However, both hypotheses do not fully explain how and why particular individuals may have different outcomes from internet use, even though they may have similar personalities or social capitals in real life. The current findings revealed that individuals’ severity of internet addiction was not directly statistically predicted by extraversion. Instead, extraversion was related to preferences for online anonymity, extraversion compensation, and online-offline integration. These three mediators, in turn, linked extraversion to internet addiction. More importantly, the previous “rich-get-richer” hypothesis and the social compensation hypothesis are not mutually exclusive, but could be more or less relevant depending on the question of whether people use the internet with the approach of online-offline integration (rather than as an escape from real life). The study also illuminated how to identify factors that could mediate connections between introversion/or extraversion and social outcomes proposed by the modified “rich-get-richer” hypothesis [28,31].

As another original contribution, our study considered the mediating role of preference for online anonymity and extraversion compensation in the theoretical model and separated online-offline integration into three specific components. The findings suggest extraversion may predict different components of online-offline integration through different paths influencing internet addiction, and longitudinal studies are needed to provide additional support for this notion. First, extraversion indirectly influenced internet addiction through the sequential mediating effects of online anonymity preference and relationship integration. Introverted individuals often more openly express themselves in anonymous environments [39], which may reflect a preference for online anonymity. Perceived anonymity may allow people to alter their offline identities [52], generating difficulties connecting with their offline friends online or meeting with their online friends offline. Therefore, their online-offline relationship integration could be hindered. Relationships are often difficult to maintain online only, without offline ties [53]. A prior study suggested that extraverted individuals may exhibit higher relationship integration as they are more likely than introverted individuals to meet their online friends face to face [54]. Our results show that online-offline relationship integration may be a protective factor mitigating against internet addiction, which is consistent with prior findings that playing online games with real-life friends may enhance individuals’ offline lives and reduce destructive virtual immersion, thereby avoiding excessive online gaming [55]. Second, extraversion compensation was found to mediate the association between extraversion and self-identity integration, suggesting introverted versus extraverted individuals are more likely to exhibit the online extraversion compensation effect, which is consistent with a previous study that revealed introverted people often behave more extraverted online [38]. In digital environments, introverted individuals may be able to shed real-world anxieties [56], experience less inhibition, and be more outgoing [40]. However, our results suggest that this kind of extraversion compensation may be related to less online-offline self-identity integration, which in turn may be associated with a greater likelihood for internet addiction. Lastly, the model indicated that social function integration was not predicted by a preference for online anonymity or extraversion compensation. However, it mediated the association between extraversion and internet addiction as anticipated. According to the “integrated-get-richer” hypothesis, the offline-first principle emphasizes that people should give higher priority to reality and use the internet as a supplement to real life. Namely, to achieve online-offline social function integration, people should use the internet more to fulfill real-life functions (e.g., school, work, or family activities) rather than to escape from real-life problems [32]. The results reveal that extraverted individuals may have a high level of online-offline social function integration, resonating with prior findings that people who spend more time engaged in entertainment are more introverted [57]. 

The study has multiple implications. First, the guidelines of online-offline integration may be promoted and act as a compass for promoting a healthy use of the internet. Integration intervention materials may focus on promoting online-offline self-identity, interpersonal relationships, and social-function integration. Second, the “double-edged sword” effect of internet features deserve our attention. While the unique features of the internet may provide more space and possibilities for individuals to promote self-disclosure, experiment with new identities, explore novel abilities, or meet new friends [58], individuals may present themselves online in ways that differ from real life [59], thus generating conflicts, impacting well-being [60], and increasing the likelihood of internet addiction [32]. It is important to increase online-offline integration by bridging them with communication and achieving a dynamic consistency involving the transfer of new possibilities to each other [32,33]. Third, prevention programs may target introverted individuals, as they may be more likely to use the internet with preference for online anonymity and exhibit compensation for extraversion, and less likely to have the internet serve their social functions. To intervene or prevent internet addiction, it may be recommended to individuals to decrease their anonymity in online communications, transfer higher levels of extraversion or communication skills rehearsed on the internet to face-to-face social environments [61], and use the internet more to promote their social, professional, or academic functioning, which are responsibilities often required in real-life social roles.

Several limitations of the current study should be noted. First, causality may not be inferred because cross-sectional data were examined. Therefore, future longitudinal research is needed to confirm the modeled mediation findings. Second, future studies should aim to enhance the proposed and tested model by identifying more factors that may influence associations between extraversion and the three components of online-offline integration. Third, extraversion may have different predictive pathways for different subtypes of internet addiction that were not distinguished in this study. For example, a study on game avatars suggested individuals’ avatar identification could predict problem gaming through self-concept clarity [62]. From this perspective, self-identity integration may be a predictor of internet gaming disorder in the model. Other studies have found that social media addiction was correlated with various aspects of interpersonal relationships (e.g., peer relationships, adolescent−parent relationships) [63,64], that may indicate a closer relationship between relationship integration and social media addiction. Thus, given that different, specific types of internet addiction may have different predictive pathways in the model, they should be further examined in the future. Considering that the supporting evidence for different hypotheses may be not definitive [29], it is also important for future studies to examine the “integrated-get-richer” hypothesis on a wider spectrum, such as to explore whether and how various proxy measures of the socially rich or poor (e.g., social anxiety, loneliness) may get “richer” or “poorer” in other psychological measures (e.g., social capital, subjective well-being) through components of online-offline integration. An additional limitation includes the lack of a formal power analysis, although a rationale for determining the samples was used and significant findings were observed. Although the current study demonstrated the importance of personality features, including extraversion and its relationship with internet addiction, the importance of other factors should not be ignored. Additional studies based on a holistic perspective, such as that described in biopsychosocial models [8], are needed to provide a more comprehensive understanding of internet addiction and the factors that influence it [65].

## 5. Conclusions

This study provides initial empirical data explaining how extraversion may influence internet addiction through online-offline integration within the framework of the “integrated-get-richer” hypothesis. The data suggest that extraversion does not directly influence internet addiction, but rather operates indirectly through mediators of preference for online anonymity, extraversion compensation, and online-offline integration. Regarding specific integration components, preference for online anonymity and relationship integration sequentially mediated the association between extraversion and internet addiction, extraversion compensation and self-identity integration also sequentially mediated that relationship, and social function integration by itself mediated the link from extraversion to internet addiction. The findings help deepen an understanding of the relationship between personality and internet addiction and may guide further studies and help develop interventions that target online-offline integration strategies for reducing problematic internet use.

## Figures and Tables

**Table 1:** Means, standard deviations, and correlations between variables (n = 428).

Variables	1	2	3	4	5	6	7	8
1. Extraversion	1							
2. Preference for online anonymity	−0.39 **	1						
3. Extraversion compensation	−0.50 **	0.19 **	1					
4. Online-offline integration	0.33 **	−0.25 **	−0.26 **	1				
5. Self-identity integration	0.26 **	−0.15 **	−0.28 **	0.74 **	1			
6. Relationship integration	0.27 **	−0.31 **	−0.18 **	0.73 **	0.36 **	1		
7. Social function integration	0.18 **	−0.07	−0.11 *	0.70 **	0.29 **	0.21 **	1	
8. Internet addiction	−0.17 **	0.15 **	0.19 **	−0.50 **	−0.38 **	−0.26 **	−0.46 **	1
M	7.15	40.14	−0.08	42.47	14.94	13.46	14.06	48.86
SD	3.14	7.18	2.93	5.26	2.27	2.55	2.46	11.73

* *p* < 0.05, ** *p* < 0.01.

**Table 2:** Bootstrapping estimated effects and 95% confidence intervals (CIs) for the mediation model.

Path	Effect	95%CI
**Direct effect**		
Extraversion → internet addiction	0.04	−0.06, 0.12
**Indirect effects**	−0.21	−0.28, −0.14
Extraversion → preference for online anonymity → internet addiction	−0.01	−0.04, 0.03
Extraversion → extraversion compensation → internet addiction	−0.04	−0.08, 0.01
Extraversion → online-offline integration → internet addiction	−0.10	−0.15, −0.05
Extraversion → preference for online anonymity → online-offline integration → internet addiction	−0.04	−0.06, −0.02
Extraversion → extraversion compensation → online-offline integration → internet addiction	−0.03	−0.05, −0.01

## Data Availability

Data collected as part of this study are available on request from the corresponding author.

## Appendix A. Preference for Online Anonymity Questionnaire

The following statements are related to your thoughts and behaviors toward your online identity usage. Please decide whether each statement applies to you using a scale with numbers corresponding to 1 = strongly disagree, 2 = disagree, 3= Neutral, 4 = agree, and 5 = strongly agree.

1. Friends I meet online know my true identity. (R)
2. The username I use on the social networking sites has nothing to do with my real identity.
3. I am used to using my real photos as avatars on social platforms. (R)
4. I often use fake gender, age or location on online platforms.
5. I like to express different opinions to others online in an anonymous way.
6. I typically use my real name on online social-networking sites. (R)
7. I mostly use my real identity in discussions of social topics on the internet. (R)
8. Compared to being anonymous, I am more accustomed to using my real name to talk about different opinions with others on the internet. (R)
9. I prefer to use online service platforms that require a real-name account. (R)
10. I use an anonymous identity to participate in social discussions on the internet.

Note: R = reverses item.
